# Live Organoid Cyclic Imaging

**DOI:** 10.1002/advs.202309289

**Published:** 2024-02-07

**Authors:** David E. Reynolds, Yusha Sun, Xin Wang, Phoebe Vallapureddy, Jianhua Lim, Menghan Pan, Andres Fernandez Del Castillo, Jonathan C. T. Carlson, Mark A. Sellmyer, MacLean Nasrallah, Zev Binder, Donald M. O'Rourke, Guo‐li Ming, Hongjun Song, Jina Ko

**Affiliations:** ^1^ Department of Bioengineering University of Pennsylvania Philadelphia PA 19104 USA; ^2^ Department of Neuroscience Mahoney Institute for Neurosciences Perelman School of Medicine University of Pennsylvania Philadelphia PA 19104 USA; ^3^ Department of Biochemistry & Molecular Biophysics Perelman School of Medicine University of Pennsylvania Philadelphia PA 19104 USA; ^4^ Center for Systems Biology Massachusetts General Hospital Boston MA 02114 USA; ^5^ Department of Medicine Massachusetts General Hospital Harvard Medical School Boston MA 02114 USA; ^6^ Department of Radiology Perelman School of Medicine University of Pennsylvania Philadelphia PA 19104 USA; ^7^ GBM Translational Center of Excellence Abramson Cancer Center University of Pennsylvania Philadelphia PA 19104 USA; ^8^ Department of Pathology and Laboratory Medicine University of Pennsylvania Philadelphia PA 19104 USA; ^9^ Center for Cellular Immunotherapies University of Pennsylvania Philadelphia PA 19104 USA; ^10^ Department of Neurosurgery Perelman School of Medicine University of Pennsylvania Philadelphia PA 19104 USA; ^11^ Department of Cell and Developmental Biology Perelman School of Medicine University of Pennsylvania Philadelphia PA 19104 USA; ^12^ Department of Psychiatry Perelman School of Medicine University of Pennsylvania Philadelphia PA 19104 USA; ^13^ Institute for Regenerative Medicine University of Pennsylvania Philadelphia PA 19104 USA; ^14^ The Epigenetics Institute Perelman School of Medicine University of Pennsylvania Philadelphia PA 19104 USA

**Keywords:** biomarker discovery, bioorthogonal click‐chemistry, glioblastoma, longitudinal monitoring, multiplexing, organoids

## Abstract

Organoids are becoming increasingly relevant in biology and medicine for their physiological complexity and accuracy in modeling human disease. To fully assess their biological profile while preserving their spatial information, spatiotemporal imaging tools are warranted. While previously developed imaging techniques, such as four‐dimensional (4D) live imaging and light‐sheet imaging have yielded important clinical insights, these technologies lack the combination of cyclic and multiplexed analysis. To address these challenges, bioorthogonal click chemistry is applied to display the first demonstration of multiplexed cyclic imaging of live and fixed patient‐derived glioblastoma tumor organoids. This technology exploits bioorthogonal click chemistry to quench fluorescent signals from the surface and intracellular of labeled cells across multiple cycles, allowing for more accurate and efficient molecular profiling of their complex phenotypes. Herein, the versatility of this technology is demonstrated for the screening of glioblastoma markers in patient‐derived human glioblastoma organoids while conserving their viability. It is anticipated that the findings and applications of this work can be broadly translated into investigating physiological developments in other organoid systems.

## Graphical abstract

A bioorthogonal click chemistry technology to display the first demonstration of multiplexed cyclic imaging of live and fixed patient‐derived glioblastoma tumor organoids. This approach enhances molecular profiling by quenching fluorescent signals from the surface and intracellular of labeled cells across multiple cycles, allowing for more efficient profiling of their complex phenotypes.

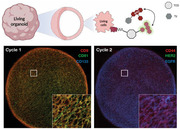

## 1 Introduction

In recent years, organoids have garnered attention for lending greater insight into human biology and disease, enhancing diagnostics and therapeutics.^[^ ^1^, ^2^, ^3^ ^]^ Given their close physiological similarities to human tissues and organs, organoids satisfy unfulfilled needs in traditional in vivo and in vitro model systems.^[^ ^4^ ^]^ Because organoids can be infected with viral and bacterial pathogens, they have been used to diagnose and treat infectious diseases like SARS‐COV‐2, respiratory syncytial virus, and influenza.^[^ ^5^, ^6^, ^7^, ^8^ ^]^ These mini‐organs have also been applied to investigate difficult‐to‐access and model conditions, like cystic fibrosis and psychiatric disorders.^[^ ^9^, ^10^, ^11^, ^12^ ^]^ Not to mention, tumor organoids have been used in cancer research to anticipate the effects of chemotherapies and improve targeted treatments due to their ability to represent numerous tumor forms.^[^ ^13^ ^]^ Gene editing techniques, such as CRISPR‐coupled single‐cell transcriptomics, have also been applied in organoid models for recording and understanding single‐cell lineage dynamics.^[^ ^14^ ^]^ Together, organoids have been used in an increasing number of applications, demonstrating their significance in science and medicine.

Image‐based approaches are critical for the spatial conservation and cellular assessment of organoids in biological and clinical applications, but their intricate structure requires the employment of more sophisticated strategies beyond traditional two‐dimensional (2D) imaging. Methods previously explored include four‐dimensional (4D) live imaging of organoids via confocal live‐cell microscopy and high‐throughput screening imaging combined with a fluorescence‐based swelling assay.^[^ ^15^ ^]^ Light‐sheet imaging has also been used to analyze whole organoids and their cellular components, achieving high‐throughput screening of 300 organoids h^−1^.^[^ ^16^, ^17^ ^]^ Despite the advances in live imaging, these methods are incapable of multiplex imaging, which is necessary to gain a complete overview of the organoid's complex phenotype. More commonly, fixed organoids are used to analyze the expression of multiple markers (*N* ≤ 4); however, this eliminates the capacity for longitudinal monitoring of the same organoid over time.^[^ ^18^, ^19^, ^20^, ^21^, ^22^ ^]^ Overall, while organoids are exponentially growing in attention, there has been a lack of focus on developing more sophisticated and rapid technologies for the multiplex and longitudinal screening of their phenotypes.

In this article, we introduce a click chemistry‐based approach for conducting multiplexed (*N* > 9) imaging on patient‐derived live brain organoids. This evolved cyclic imaging method has already yielded significant discoveries in various biological components, including tissues, cells, and extracellular vesicles.^[^ ^23^, ^24^, ^25^, ^26^, ^27^ ^]^ By implementing this technique on live and fixed organoids, we aim to address the existing challenges in multiplexing analysis. Leveraging the tetrazine/trans‐cyclooctene (Tz/TCO) reaction's remarkable ultrafast kinetics and bioorthogonal properties, we have seamlessly integrated it into immunostaining techniques. As a result, our method allows for the simultaneous capture of multiple biological processes, presenting a valuable tool for advancing research on live organoids and gaining deeper insights into their intricate biology. One illustrative application of our approach involves screening for specific and nonspecific glioblastoma markers in human brain organoids while closely monitoring their viability. Through this multiplex analysis, we can effectively observe the relative co‐localization and abundance of these glioblastoma markers. Overall, we anticipate that the findings and applications of this work will extend beyond brain organoids, providing valuable insights into physiological developments across diverse organoid systems.

## 2 Results

### 2.1 Live and Fixed Organoid Staining and Quenching

**Figure 1:**
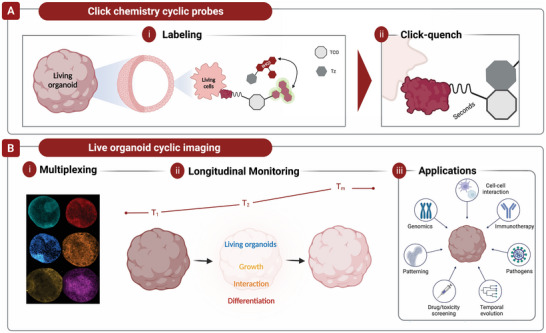
Schematic of live organoid cyclic imaging. A) Living organoids and their individual living cells are stained with antibody TCO‐FL. After imaging, the signal is rapidly quenched with BHQ3‐Tz. B) With the click chemistry cyclic probes, multiplexing, and longitudinal monitoring can be executed.

**Figure 2:**
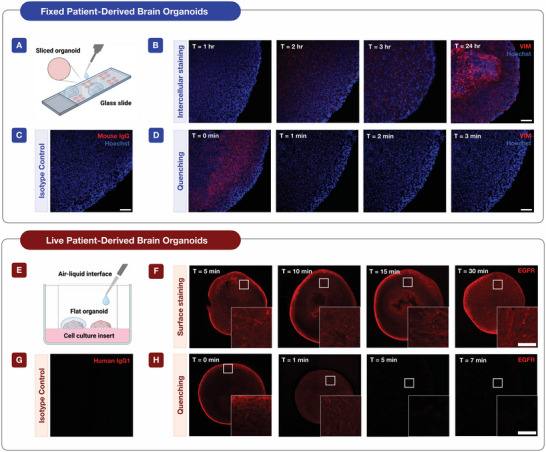
Time points for staining and quenching intracellular and surface proteins. A) Schematic for staining, washing, and imaging fixed sliced organoids. B) Intracellular staining time validation for fixed brain organoids. Samples were incubated with VIM‐TCO‐FL and imaged at different time points. C) Mouse IgG isotype control for VIM staining (24‐h incubation) (Scale bar = 100 µm). D) Intracellular quenching time validation for fixed brain organoids. Samples were incubated with BHQ3‐Tz and imaged at different time points (Scale bar = 100 µm). E) Schematic for staining, washing, and imaging live flat organoids. F) Surface staining time validation for live brain organoids. Samples were incubated with EGFR‐TCO‐F and imaged at different time points. G) Human IgG1 isotype control for EGFR staining (5 min incubation) (Scale bar = 100 µm). H) Surface marker quenching time validation for live brain organoids. Samples were incubated with BHQ3‐Tz and imaged at different time points (Scale bar = 100 µm).

The technology is based on the idea of rapidly and effectively quenching antibody‐conjugated fluorescent dyes of varying wavelengths without obstructing native biochemical processes. This process is achieved using a modular linker that connects the fluorochromes and antibodies with an embedded TCO molecule (antibody‐TCO‐fluorophores) for clicking with Tz conjugated to a quencher (**Figure** **1**A). Based on our previous work, the appropriate quencher that was chosen for our application was the black hole quencher 3 (BHQ3). BHQ3‐Tz has been reported to accelerate the interaction between the Tz‐TCO click reaction, achieving immediate (seconds) and highly efficient (>95%) quenching of the dye's fluorescence. This prevents time‐consuming cleavage or bleaching steps and permits rapid cellular analysis.^[^ ^25^, ^27^ ^]^ To validate this click chemistry‐based ultra‐fast cyclic imaging method on organoids, we chose patient‐derived glioblastoma organoids as our model system (Figure 1B). These patient‐derived tissue samples were prepared as described previously by Jacob et al.^[^ ^28^, ^29^ ^]^ from resected surgical tissues maintained with routine manual dissection and orbital shaking. To determine the optimal incubation and quenching times for intracellular and surface protein staining with the antibody‐TCO‐fluorophores (Ab‐TCO‐FL), the conjugate was incubated at varying time points with the organoids. For intracellular protein staining, the organoids were fixed and cryosectioned at 25 µm thickness on glass cover slides (**Figure** **2**A). Subsequently, these samples were incubated with a vimentin (VIM) targeting antibody and imaged at different time points (1, 2, 3, and 24 h) (Figure 2B). For the 0–3 h samples, they were kept at room temperature, while the overnight sample was maintained at 4°C. While the fluorescence signal began appearing at 2 h, complete saturation was not observed until overnight at 4°C. These findings are in agreement with previous findings, in which they report that VIM has fluorescence saturation after overnight incubation at 4°C.^[^ ^30^ ^]^ An isotype control (mouse IgG) for the VIM antibody, which was conjugated with an NHS‐647FL, confirmed that the validated VIM antibody signal was real (Figure 2C). For quenching, the BHQ3‐Tz probe was added to the overnight 4°C sample and imaged at several time points (0, 1, 2, and 3 min) (Figure 2D). Complete quenching was observed at 1 min, but to ensure the complete removal of the residual fluorescence signal, 2 min is recommended. Thus, for optimal organoid intracellular protein cyclic imaging, Ab‐TCO‐FL and BHQ3‐Tz incubations are recommended overnight at 4°C and for 2 min at room temperature, respectively.

For live imaging, we used a liquid interface system to image the organoids. The organoids were prepared by plating single glioblastoma organoids on a culture insert for 1 day prior to imaging (Figure 2E). Each sample was incubated with an epidermal growth factor receptor (EGFR) targeting antibody at varying time points (5, 10, 15, and 30 min) (Figure 2F). Between 5 and 30 min, there were no significant differences in fluorescence intensity. An isotype control (Human IgG1) for the EGFR antibody confirmed that the validated EGFR signal was real (Figure 2G). For quenching, we incubated the samples with the BHQ3‐Tz at several time points (0, 1, 5, and 7 min) (Figure 2H). Observations indicate that the quencher eliminated the signal on the periphery of the organoids around 1 and 2 min, and then penetrated the core around 5 min. We believe the less effective quenching efficiency, comparatively to the fixed organoids, is attributed to the loss of reagent into the media reservoir that is needed for live organoid culture. Therefore, to compensate for this limitation in our live imaging and culturing system, complete quenching can be expected at 7 min. The whole organoids in suspension achieved complete quenching within 1 min (Figure S1, Supporting Information). Together, with these validated staining and quenching time points, multiplexing in fixed and live organoids could be performed.

### 2.2 Multiplexed Imaging in Fixed Patient‐Derived Glioblastoma Organoids

For the multiplexed assessment of human glioblastoma organoids, a combination of specific and nonspecific glioblastoma biomarkers was chosen for our panel. For glioblastoma, there remains limited reported literature on commonly expressed surface biomarkers. Some of those commonly reported markers, though, include CD15, CD44, CD70, CD133, and EGFR.^[^ ^31^, ^32^, ^33^ ^]^ Through primary and secondary antibody incubation with the glioblastoma organoids, only a handful of the surface markers were well expressed, like CD44, CD133, and EGFR. To compensate, we validated and included common surface proteins found in most cancer types, like CD9, CD81, and human epidermal growth factor receptor 2 (HER2). Based on the heterogeneity of their relative expressions, we found their inclusion appropriate in the fixed multiplexed panel. Because we also wanted to demonstrate the application of our click‐based chemistry technology with intracellular protein targets, we included VIM, alpha‐2‐Macroglobulin (A2M), and carbonic anhydrase 9 (CA9). Based on previous findings, VIM is reported to be expressed 11‐fold greater in glioblastoma than in non‐tumor brain tissue.^[^ ^34^ ^]^ In addition, A2M and CA9 have also been found to be up‐regulated in glioblastoma tissue.^[^ ^35^, ^36^ ^]^ Before running the full panel, we validated each antibody with primary and secondary antibody staining (Figure S2, Supporting Information). Then, we tested the staining and quenching efficiency of each antibody with TCO‐FL and BHQ3‐Tz, respectively, using the optimal conditions discovered in the previous section (Figure S3, Supporting Information). After validation, we screened a combination of nine markers in the glioblastoma organoids, requiring three cycles.

**Figure 3:**
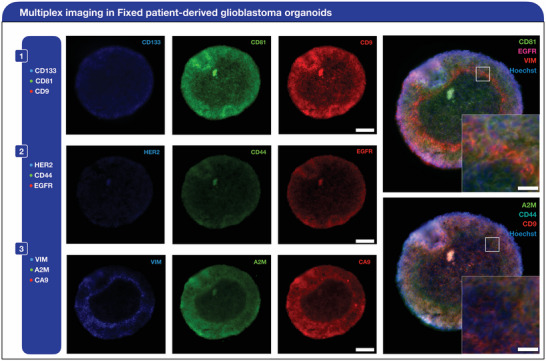
Multiplexed analysis in patient‐derived glioblastoma organoids. A combination of nine different markers was stained, quenched, and imaged. This mixture of markers was divided into three separate cycles: cycle 1 (CD133, CD81, and CD9), cycle 2 (HER2, CD44, and EGFR), and cycle 3 (VIM, A2M, and CA9) (Scale bar = 25 µm). Composite images, including hoechst and 1 target from each cycle, are included on the right to showcase the co‐localization of surface and intracellular protein expression (Scale bar = 25 µm).

Based on the cyclic imaging, we observed that the relative abundance of each protein is heterogeneous (**Figure** **3**). For instance, for the surface panel, CD9 and CD81 had the highest expression. Podergajs et al. and Wang et al. reported that CD9 is a glioblastoma biomarker that is relevant for the maintenance of glioblastoma stem cells.^[^ ^37^, ^38^ ^]^ Other groups, like Jennrich et al., claim that CD9 and CD81 positive EVs are used as biomarkers for glioblastoma, which corroborates their relatively high expression on their parent cells.^[^ ^39^ ^]^ For the more specific glioblastoma biomarkers, like CD44, CD133, and EGFR, their expression was comparatively lower. However, relative to non‐specific glioblastoma surface markers, like HER2, their signal remains considerably more apparent. For the intracellular markers, the relative expression remained closely similar. However, the localization for VIM was observed to be more apparent on the periphery of the sliced sample. Nevertheless, their high expression supports their up‐ regulation in glioblastoma tissue. With our bioorthogonal click‐based chemistry technology, we were able to screen a combination of nine different surface and intracellular markers in the same organoid, showcasing the co‐localization of their relative expression. To demonstrate the cyclic probes and quenching do not interfere with staining on the succeeding cyclic, we prepared two separate samples and incubated one sample with a HER2 cyclic imaging probe. Following incubation, the sample was quenched with the BHQ3‐Tz probe. Then, both samples were incubated with a primary EGFR antibody, followed by a complementary secondary antibody. Based on our results, the mean fluorescence intensity remained similar between both samples, indicating that the second immunostaining cycle is unaffected by the previous cycle (Figure S4, Supporting Information). Because organoids are dynamic mini‐organs, their individual cells are constantly proliferating, differentiating, and interacting with one another. Thus, our rapid cyclic imaging approach is warranted for profiling and assessing their progression and development over time.

### 2.3 Live Organoid Multiplexed and Viability Analysis

**Figure 4:**
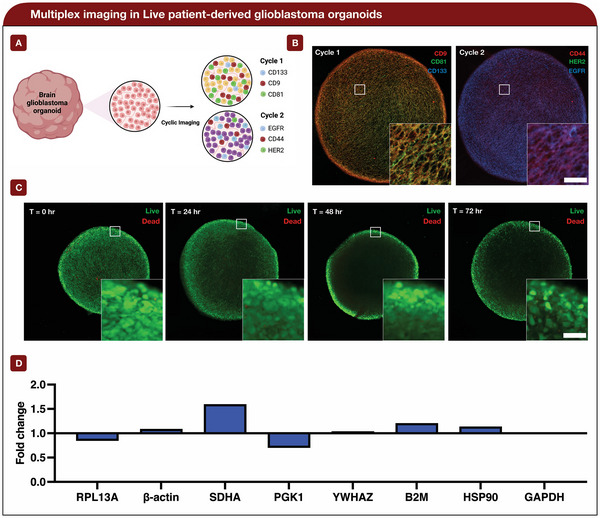
Multiplex imaging and viability assessment in live patient‐derived glioblastoma organoids. A) Schematic of live cyclic imaging panel for patient‐derived brain organoids. B) Multiplex imaging in live patient‐derived glioblastoma organoids. A combination of six different surface markers was stained, quenched, and imaged. This mixture of markers was divided into two separate cycles: cycle 1 (CD9, CD81, and CD133) and cycle 2 (CD44, HER2, and EGFR) (Scale bar = 50 µm). C) A live/dead assessment was performed on the same organoids to evaluate for organoid viability (live (Green) & dead (Red)). Time stamps for 0, 24, 48, and 72 h are reported (Scale bar = 50 µm). D) Housekeeping gene assessment 24 h post cyclic imaging versus no cyclic imaging (negative control). Fold change was calculated using 18s rRNA as a housekeeping gene. Three replicate wells were used for each gene across both samples (*n* = 3).

Multiplexed analysis has yet to be demonstrated in live organoids, thus, we prove its first application in patient‐derived glioblastoma organoids. From our fixed multiplexed panel, we targeted the same group of surface proteins (CD9, CD81, CD133, CD44, HER2, and EGFR) ( **Figure** **4**A). Similar to our previous staining and quenching validation in live organoids, we used the same experimental setup for imaging and culturing the live glioblastoma organoids. For our first cycle, we stained for CD9, CD81, and CD133. Based on the overlayed image, CD9 and CD81 are highly expressed, while CD133 is minimally expressed across the organoid sample. These similar observations were reported in our fixed multiplexed organoid analysis. For the second cycle, CD44, HER2, and EGFR proteins were targeted. Similar to our fixed multiplexed analysis, only CD44 and EGFR were observed to have expression (Figure 4B). Together, with our live multiplexed assessment of the glioblastoma organoids, we are able to observe the relative co‐localization and expression of different proteins with our click‐based chemistry technology.

To confirm that the organoid's viability was not affected by our staining and quenching process, a live/dead assay was performed with the organoids at varying time points after cyclic imaging. Between 0 and 72 h, we did not observe any significant changes in the organoid's health (Figure 4C). In other words, the observed number of dead cells remained negligible. For our control, which did not include cyclic imaging, the number of dead cells was similar to the sample with cyclic imaging (Figure S5, Supporting Information). To gain a more quantitive understanding of organoid viability, a cell proliferation assay was performed 24 h post cyclic imaging. Based on the assay, the cyclic imaging and negative control absorbance readings were 0.49 and 0.46, respectively. In this case, there were no significant differences between the cyclic imaging and negative control samples. The large error bars can be attributed to the organoids’ size and shape heterogeneity (Figure S6, Supporting Information). Additionally, a combination of nine different housekeeping genes was screened between both sets of samples.^[^ ^40^, ^41^, ^42^ ^]^ Based on qPCR, there remained no significant differences between each gene, which was confirmed through an unpaired *t*‐test (not statistically significant *p* = 0.82). After calculating fold change, using 18s rRNA as a housekeeping gene, each gene remained very close to 1. This indicates there is negligible fold change between cyclic imaging and no cyclic imaging (Figure 4D). Between both samples, though, we observed the core of the organoids becoming less dense over time. We believe this is attributed to the migration of the cells to the outer surface of the organoid, as there may be a stronger concentration of nutrients near the surface. Nonetheless, we can confirm that the overall general health of the organoid remains undisturbed. This similar reporting was recorded by *Ko* et al., who demonstrated that our live cyclic imaging approach does not interfere with single‐cell viability, as well as differentiation.^[^ ^25^ ^]^ With our multiplexed and viability analysis, we demonstrated the application of our click‐based chemistry approach for screening multiple proteins on live organoids and proved their health remains undisturbed.

## 3 Discussion

Organoids have enhanced our knowledge of disease pathology and treatment options, enabling their biological and clinical applications to expand rapidly. Because organoids are exponentially growing in interest, spatiotemporal imaging tools are warranted. While prior imaging approaches, such as 4D live imaging and light‐sheet imaging have provided significant clinical insights, they lack the combination of cyclic and multiplexed analysis. To overcome these challenges, we applied our cyclic imaging method that employs bioorthogonal click chemistry to stain and rapidly quench fluorescent signals in multiple cycles. This technique allows us to profile many proteins in patient‐derived organoids using commercially available antibodies. Otherwise, we would have to genetically modify or fluorescently tag proteins of interest for live cell imaging, which is not possible with patient samples. To demonstrate this proof‐of‐principle, we applied this approach to screen glioblastoma markers in live and fixed patient‐derived brain organoids. Based on our findings, our cyclic imaging method has the potential to transform our understanding of organoids and their role in disease pathology and treatment. By enabling multiplexed analysis, we can gain a deeper understanding of organoid biology and accelerate the development of new clinical applications.

While the technology has its advantages, there remain limitations. For instance, only a proteomic profile can be assessed with our click‐based chemistry approach. To gain a more comprehensive overview of the current organoid state, a multiomic approach may be warranted. Not to mention, intracellular staining requires long incubations and cannot be performed in live organoids. These limitations, though, are not attributed to the technology but the organoids themselves. In the future, we aim to continue to innovate our click‐based chemistry approach to improve its versatility. Nevertheless, our findings have significant ramifications for the development of new treatments for glioblastoma and other disorders. For instance, we can create more efficient and tailored therapies that enhance patient outcomes by improving our comprehension of the interactions between engineered immune cells and cancer cells in the setting of organoids. Overall, our research has opened exciting new directions for studying the intricate biology of organoids and improving our capacity for developing new treatments.

## 4 Experimental Section

### Antibodies

Bovine serum albumin (BSA)‐free antibodies were purchased from companies and stored under manufacturer guidelines. Optimization of immunofluorescence staining and quenching methods was performed using Cetuximab (anti‐EGFR antibody, Selleckchem) and Vimentin (anti‐Vimentin antibody, CDI Laboratories). All antibodies used for imaging were tabulated in Figure S7 (Supporting Information).

### Cyclic Imaging Probes

NHS‐TCO‐FL and BHQ3‐Tz were synthesized as described by Ko et al.^[^ ^27^ ^]^ To conjugate antibodies with NHS‐TCO‐FL, the antibodies were buffer exchanged into 0.1 m, pH 8.4 sodium bicarbonate buffer using a 40k Zeba Column (Thermo Fisher Scientific, 87765). Following the buffer exchange, the antibodies were incubated with a tenfold molar excess of NHS‐TCO‐FL linker for 30 min at room temperature (RT) on a shaker in the dark. Removal of excess unreacted dye molecules was performed twice using a 40k Zeba column (equilibrated with PBS). The absorbance spectra of the conjugated antibodies were measured using the NanoDrop1000 (Thermo Fisher Scientific) to determine the degree of labeling (DOL). Known extinction coefficients of the dye, IgG antibody, and standard correction factor for the dye absorbance at 280 nm (CF 280) were accounted for in the DOL calculations. The conjugated antibodies were stored in the dark at 4°C in PBS.

### Isotype Control Conjugation

For conjugating isotype antibodies to NHS‐fluorophores, human IgG1 (BioXcell) and mouse IgG (Thermo Fisher Scientific), the antibodies were first buffer exchanged into 0.1 m, pH 8.4 sodium bicarbonate buffer using a 40k Zeba Column. The antibodies were then incubated with a tenfold molar excess of 647 NHS‐Ester (Click chemistry tools; 1344‐1) for 30 min at RT on a shaker. The excess linker was removed with a 40k Zeba column (equilibrated with PBS) twice after incubation. The absorbance spectra of the conjugated antibodies were measured using the NanoDrop1000.

### Organoid Extraction and Culture

Glioblastoma organoids were generated as described previously by Jacob et al.^[^ ^28^, ^29^ ^]^ Organoid information, including patient ID, age, sex, and other content are included in Figure S8 (Supporting Information).

### Organoid Fixation

For generation of fixed tissue, organoids were placed in 4% formaldehyde (Polysciences; 18814‐10) for 1 h at RT with gentle rotation, and then washed 2X with PBS. Organoids were then cyroprotected by incubation in 30% sucrose in PBS overnight at 4 °C, and subsequently placed in a plastic cyro‐mold to be frozen in tissue freezing medium (General Data; 1518313) at −80 °C. Organoid samples were then sectioned (25 µm) with a cryostat (Leica; CM3050S) and stored at −20 °C until immunohistological analysis.

### Live Organoid Preparation and Culture

Cultured glioblastoma organoids were prepared for live imaging by direct placement onto an air‐ liquid interface culture (Millicell Cell Culture Insert, 0.4 µm; PICM01250) at least 24 h prior to imaging. Culture inserts were placed into six‐well plates containing 1.2 mL of glioblastoma organoids medium.^[^ ^28^, ^29^ ^]^

### Live Immunostaining and Fluorophore Quenching

Prior to immunostaining, the brain organoids were rinsed with PBS. Then, 5 µg mL^−1^ of the Ab‐ TCO‐FL probes, diluted in 1% BSA‐PBS, were added to the sample and incubated for 10  min at RT. The samples were then rinsed with PBS three times post‐incubation. Imaging was performed using a Zeiss LSM 810 or Zeiss LSM 710 confocal microscope (Zeiss) using 10x and 20x objectives with Zen 2 software (Zeiss). For fluorophore quenching, 10 µm of BHQ3‐Tz diluted in PBS was added to the sample. Post‐quench imaging was taken 10 min after the introduction of the quencher. Although the fluorescence signal removal was characteristically completed within 7 min, a routine interval was used of 10 min to ensure the complete removal of any residual fluorescence signal. To prepare for the next cycle of immunostaining, the sample was washed three times with PBS to remove any excess quencher that will disrupt the subsequent immunostaining. Live organoid imaging in Figures 2 and 4 was derived from patient UP‐10006.

### Fixed Immunostaining and Fluorophore Quenching

Prior to immunostaining, the organoid sections were air‐dried for 10 min at RT. Next, they were rehydrated using PBS with 0.1% Tween 20 (PBST) for a duration of 1 h. Following that, the sections were subjected to three consecutive washes with PBST, with each wash lasting 10 min. To prevent nonspecific binding and facilitate permeabilization, the sections were then treated with a solution of PBST containing 5% BSA and 0.3% Triton X‐100 (Sigma; X100) for 1 h at RT. Subsequently, a cocktail of Hoechst (Thermo Fisher Scientific; H3570) (1:10 000 dilution) and 5 µg mL^−1^ of the Ab‐TCO‐FL probes in 1% BSA‐PBS were added to the sample. For surface and intracellular markers, incubations were for 10 min at RT and overnight at 4°C in the dark, respectively. The samples were rinsed with PBST three times post‐incubation. Imaging was performed using an Olympus Model IX83 Inverted Fluorescence Microscope. For fluorophore quenching, 10 µm of BHQ3‐Tz diluted in PBS was added to the sample. Although the fluorescence signal removal was characteristically completed within 30 s, we used a routine interval of 2 min to ensure the complete removal of the residual fluorescence signal. To prepare for the next cycle of immunostaining, the sample was washed three times with PBST to remove any excess quencher that will disrupt the subsequent immunostaining. Fixed organoid imaging in Figures 2 and 3 was derived from patients UP‐9101 and UP‐9096, respectively.

### Organoid Viability Assessment

Organoid viability after staining and quenching was performed with a live/dead cell double staining kit (Sigma Aldrich; 04511‐1KT‐F). Organoid samples were incubated with calcein AM and propidium iodide solutions at 10 µm in PBS for 15 min at 37 °C.

### Cell Proliferation Assay

Live cultured glioblastoma organoids underwent one cycle of staining and quenching. After 24 h post cyclic imaging, organoids were incubated with a CellTiter 96 AQueous Non‐ Radioactive Cell Proliferation Assay (MTS) kit (Promega; G5421) for 1 h at 37 °C. After incubation, the plate was transferred to a GloMax Discover Microplate Reader (Promega; GM3000). The samples were shaken (duration: 10 s; cycles per minute: 300 cycles; type: linear motion; and shaking diameter: 2 mm) and had their absorbance measured at 490 nm. Organoid gene assessment was derived from patients UP‐9121.

### Housekeeping Gene Assessment

Live cultured glioblastoma organoids underwent one cycle of staining and quenching. After 24 h post cyclic imaging, RNA was extracted from the organoids with Trizol Reagent (ThermoFisher; 15596018), labeled with GlycoBlue Coprecipitant (ThermoFisher; AM9515), and treated with 2U of TURBO DNase (ThermoFisher; AM1907) at 37 °C for 30 min. DNase in activation Reagent was then added to inactivate the TURBO DNase at 0.1 volume for 5 min at RT. The iScript cDNA Synthesis Kit (BioRAD; 1708890) was used for RT‐PCR. With this kit, the cell RNA was mixed with its reagents and run in a SimpliAmp Thermal Cycler (Applied Biosystems) as per the manufacturer's protocol. The qPCR master mix includes PowerTrack SYBR Green Master Mix (Applied Biosystems; A46109), nuclease‐free water, and forward and reverse primers (Integrated DNA Technologies). 10 µL of the qPCR master mix was added to each well. For every qPCR experiment, 40 cycles were run using a QuantStudio 3 Real‐ Time PCR machine (Applied Biosystems). Organoid gene assessment was derived from patients UP‐9121.

### Statistics

Statistical analyses and line fitting were performed in GraphPad Prism 10. Data were used “as is” and was not pre‐processed. Data were presented as mean fold change for qPCR, and mean ± standard deviation for signal comparison and cell proliferation assay studies. Sample sizes (*n*) are included in figure captions. FIJI (ImageJ) software was used for all fluorescence quantification and image preparation.

## Conflict of Interest

The authors declare no conflict of interest.

## Author Contributions

D.E.R. and J.K. were responsible for the conception, design, and interpretation of the experiments and wrote the manuscript. D.E.R, Y.S., X.W., P.V., J.L., and M.P. performed experiments under the supervision of G.L.M, H.S., and J.K. A.F.C., J.C.T.C., and M.A.S. prepared chemical probes. M.N., Z.B., and D.M.R. provided tissue samples for organoid isolation and culture. All participated in data discussion and interpretation.

## Supporting information

Supporting Information

## Data Availability

The data that support the findings of this study are available from the corresponding author upon reasonable request.
