# Supplementary material for: Live Organoid Cyclic Imaging
Source: Adv Sci (Weinh). 2024 Feb 7;11(14):2309289. doi: 10.1002/advs.202309289 (PMC11005682; doi:10.1002/advs.202309289)
Supplement: Supplementary file 1 — Supporting Information [file ADVS-11-2309289-s001.pdf]

ADVANCED SCIENCE
Open Access

Supporting Information

for *Adv. Sci.*, DOI 10.1002/advs.202309289

Live Organoid Cyclic Imaging

*David E. Reynolds, Yusha Sun, Xin Wang, Phoebe Vallapureddy, Jianhua Lim, Menghan Pan, Andres Fernandez Del Castillo, Jonathan C. T. Carlson, Mark A. Sellmyer, MacLean Nasrallah, Zev Binder, Donald M. O'Rourke, Guo-li Ming, Hongjun Song and Jina Ko**

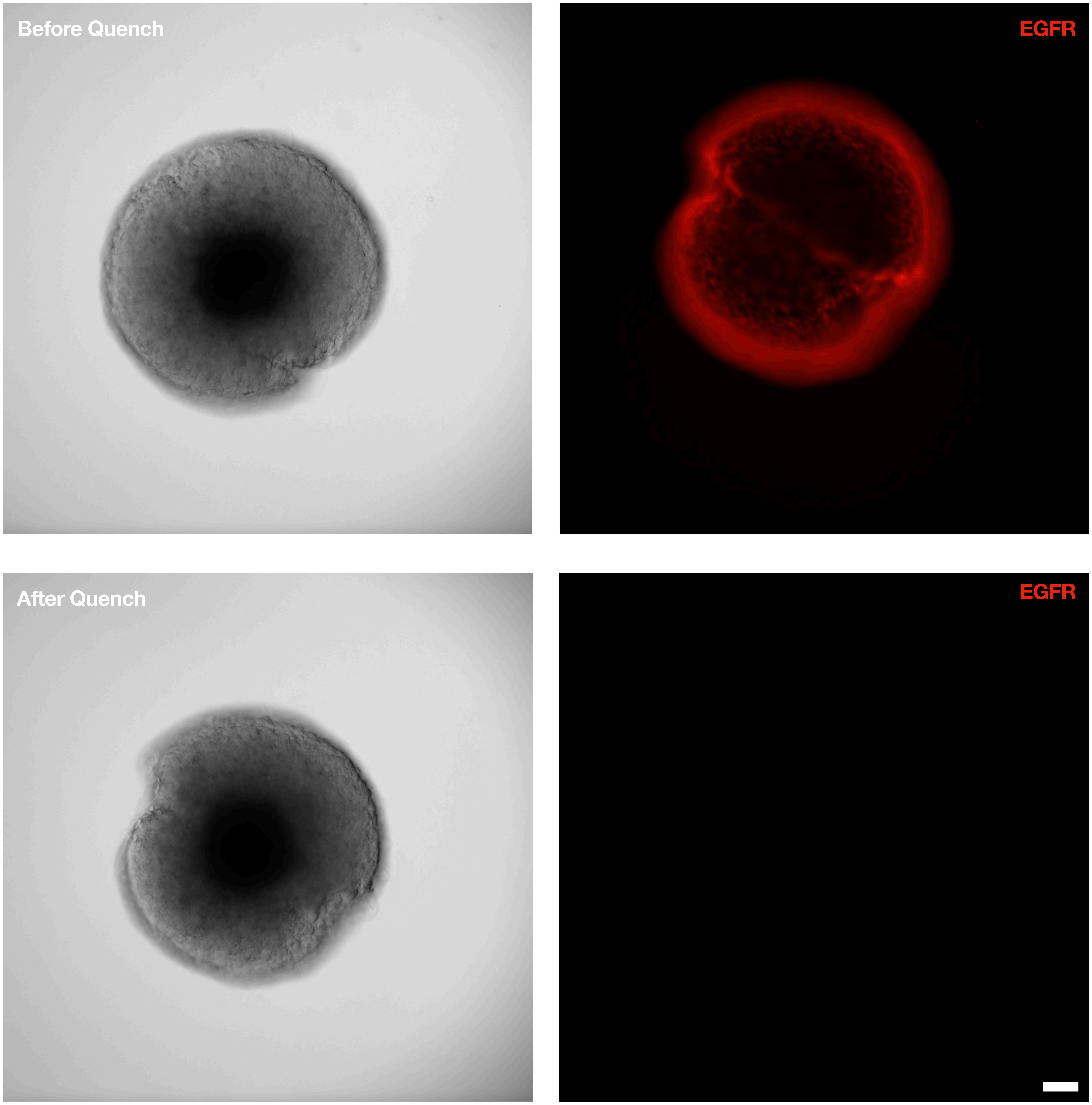

Figure S1. Whole patient-derived glioblastoma organoid staining and quenching. Glioblastoma organoids were incubated with α-EGFR-TCO-F and BHQ3-Tz probes for 5 and 1 minute, respectively. (Scale bar = 100 μm)

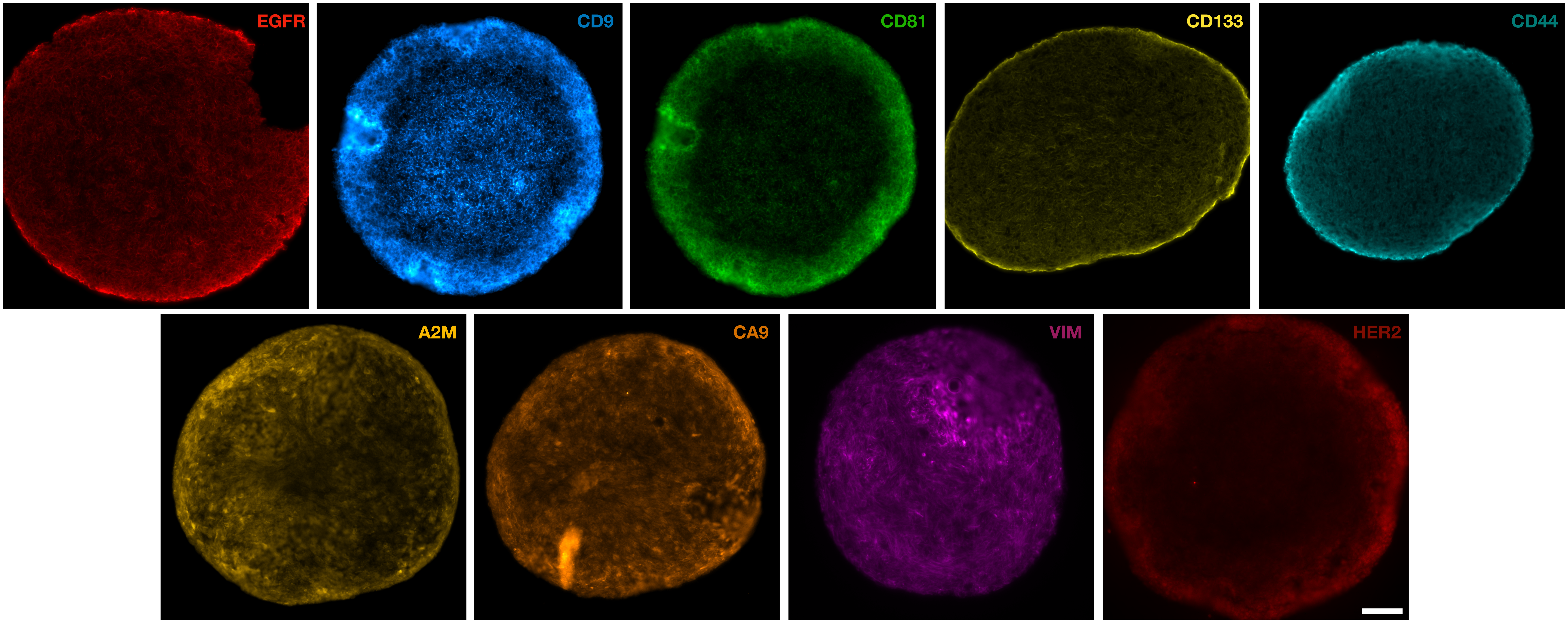

Figure S2. Primary and secondary antibody staining validation for patient-derived glioblastoma organoids. (Scale bar = 100 µm)

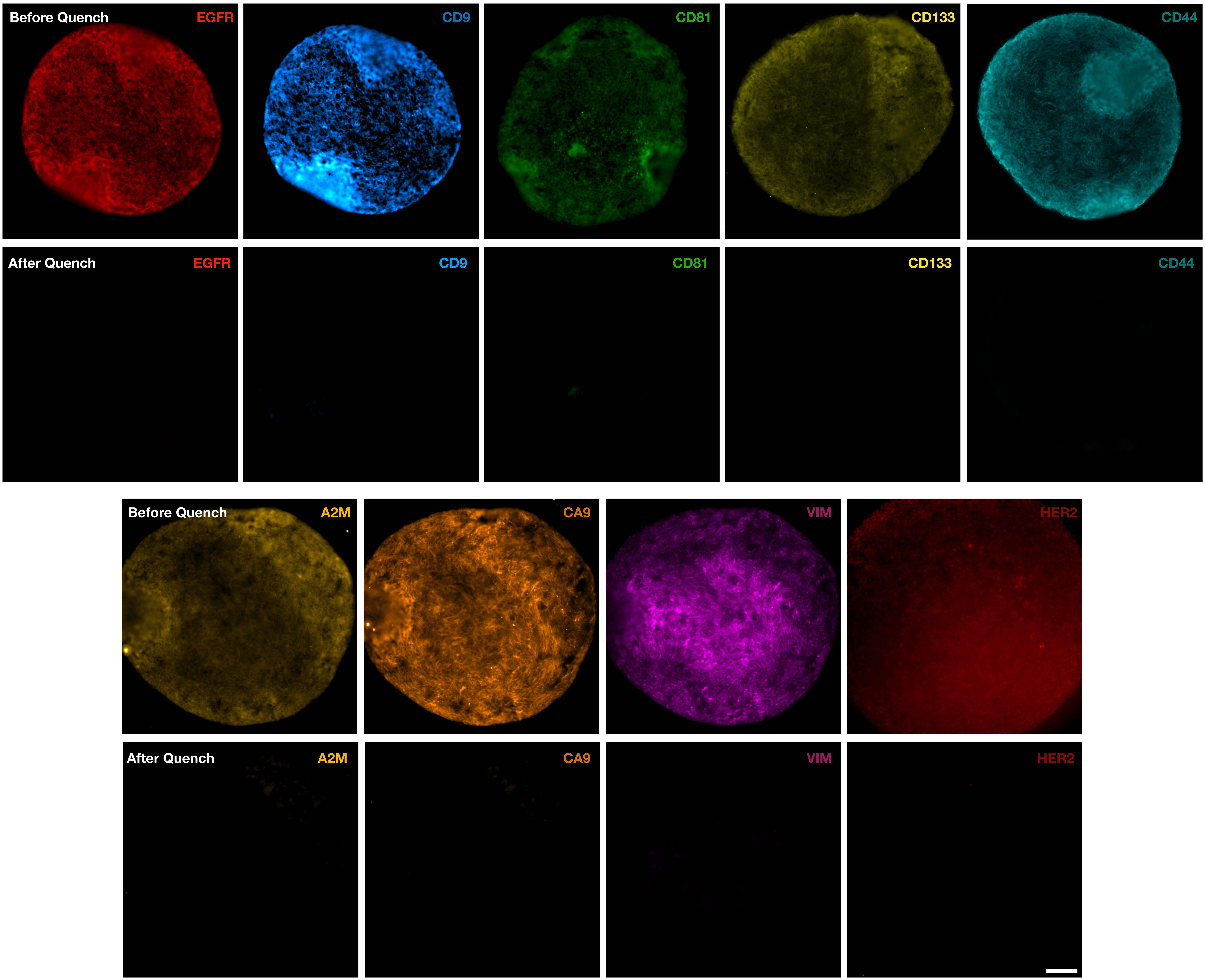

Figure S3. Antibody-TCO-F staining and BHQ3-Tz quenching validation for patient-derived glioblastoma organoids. (Scale bar = 100 μm)

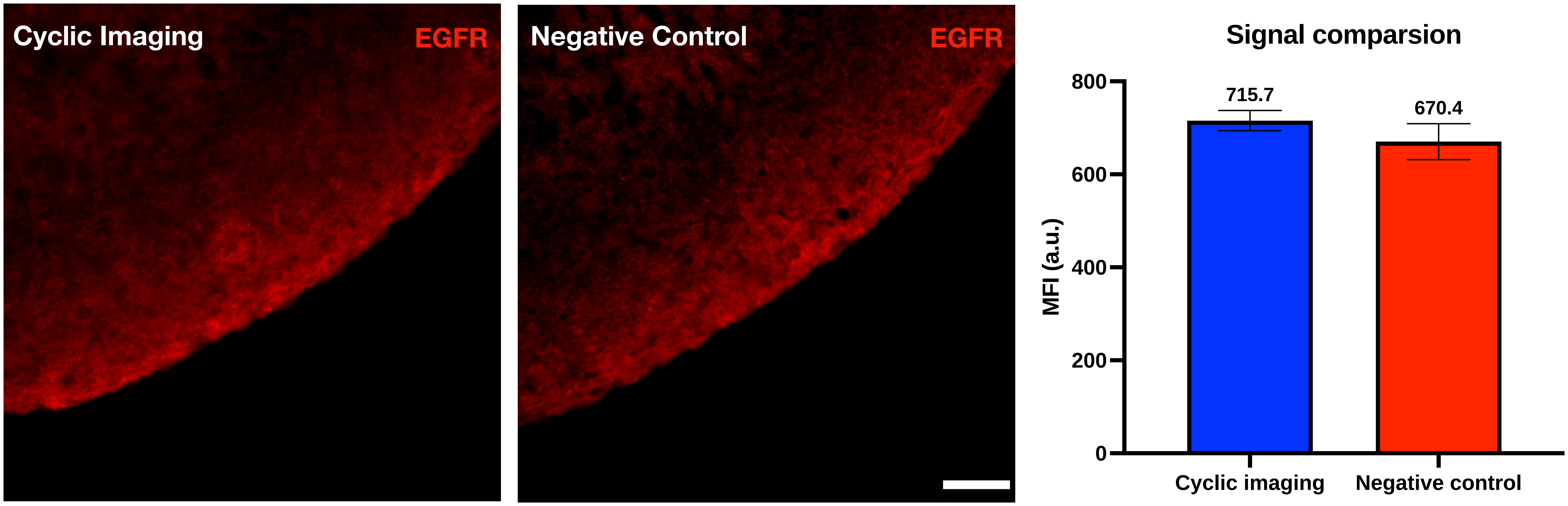

Figure S4. Signal comparison between cyclic imaging and negative control. Three images were quantified for each condition (n=3). (Scale bar = 100 μm)

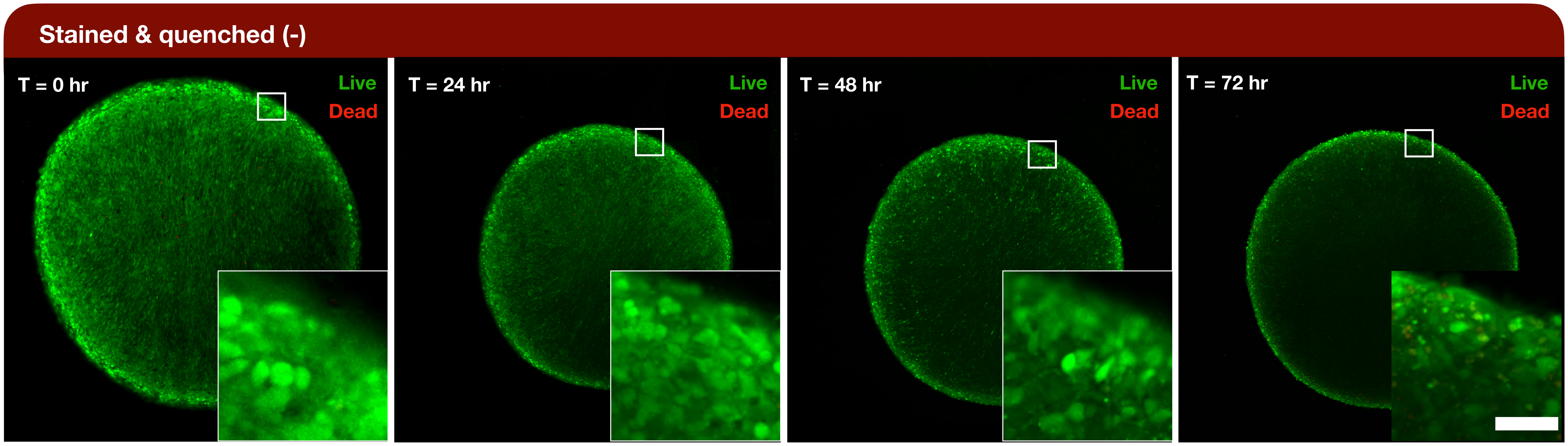

Figure S5. Live/dead assessment in stained and quenched negative (-) samples over 72 hours. (Scale bar = 50 μm)

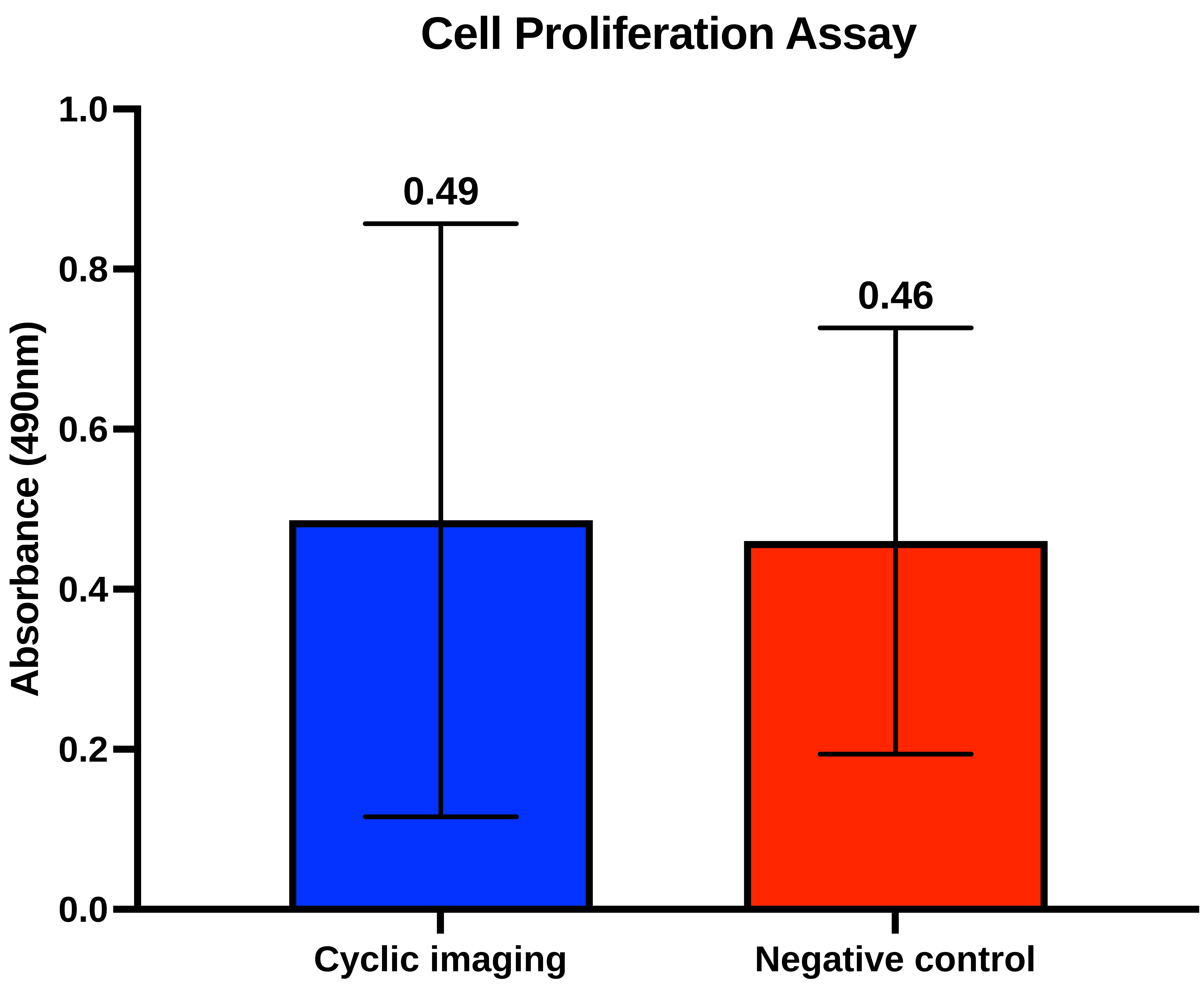

Figure S6. Cell proliferation assay for cyclic imaging and negative control. Six samples were quantified for each condition (n=6).

Target Protein	Clone	Host species	Vendor	Catalog #	Degree of labeling	Fluorophore
EGFR	Cetuximab	Human	Selleckchem	A2000	2.7 - 3.0	AF647
						AF555
Human IgG1	N/A	Human	Bio X Cell	BE0297	3.0	AF647
VIM	AB8	Mouse	CDI Laboratories	MAB60140	1.8	AF647
Mouse IgG	N/A	Mouse	ThermoFisher	02-6502	3.0	AF647
CD9	HI9a	Mouse	Biolegend	312102	1.6	AF555
CD44	IM7	Rat	Bio X Cell	BE0039	5.2	AF488
CD81	JS-81	Mouse	BD Biosciences	555675	2.9	AF488
CD133	Polyclonal	Rabbit	Proteintech	18470-1-AP	2.2	AF647
HER2	7.16.4	Mouse	Bio X Cell	BE0277	1.5	AF647
A2M	HX730.1.1A8	Mouse	CDI Laboratories	MAB576027	2.4	AF488
CA9	HX413.1.1F7	Mouse	CDI Laboratories	MAB22471	3.1	AF555

Figure S7. Antibody Table List.

Patient ID	Age	Sex	De novo/Recurrent	Histologic diagnosis	MGMT methylation	IDH status
**UP-9096**	65	M	Recurrent	Glioblastoma (CNS WHO Grade 4)	Not detected	WT
**UP-9101**	45	M	Recurrent	High-grade glioma (CNS WHO Grade 3/4)	Positive*	WT
**UP-9121**	64	M	Primary	Glioblastoma (CNS WHO Grade 4)	Not detected	WT
**UP-10006**	83	M	Primary	Glioblastoma (CNS WHO Grade 4)	Positive	WT

*Not tested on current specimen but positive on original resection

Figure S8. Patient Information Table.
